# Shorter Time to Full Preterm Feeding Using Intact Protein Formula: A Randomized Controlled Trial

**DOI:** 10.3390/ijerph16162911

**Published:** 2019-08-14

**Authors:** Maria Elisabetta Baldassarre, Antonio Di Mauro, Margherita Fanelli, Manuela Capozza, Jennifer L. Wampler, Timothy Cooper, Nicola Laforgia

**Affiliations:** 1 Neonatology and Neonatal Intensive Care Unit, Department of Biomedical Science and Human Oncology, University of Bari “Aldo Moro”, Policlinico, Piazza G. Cesare 11, 70124 Bari, Italy; 2 Medical Statistics, Department of Interdisciplinary Medicine, University of Bari “Aldo Moro”, 70124 Bari, Italy; 3 Mead Johnson Nutrition, Department of Medical Affairs, Evansville, IN 47721, USA

**Keywords:** infant, premature, infant formula, enteral nutrition

## Abstract

Background: This study was carried out to evaluate enteral feeding advancement and tolerance in preterm infants receiving one of two marketed formulas: intact protein preterm formula (IPF) or extensively hydrolyzed formula (EHF) for the first 14 feeding days. Methods: Primary outcome was days to full enteral feeding (≥140 mL/kg/day). Per protocol analyses included the following: all participants who met study entrance criteria and completed study feeding (primary) and those who received ≥75% enteral intake from study formula (subset). Mothers were encouraged to provide their breast milk. Results: Of the 65 enrolled (IPF: *n* = 32; EHF: *n* = 33), 60 completed study feeding per protocol (IPF: *n* = 30; EHF: *n* = 30), 37 (62%) received predominantly breast milk, and 23 (38%) received ≥75% study formula intake (IPF: *n* = 11; EHF: *n* = 12). No group differences were detected in tolerance measures. No necrotizing enterocolitis (NEC) was reported. Median time to achievement of full enteral feeding was significantly shorter for the IPF vs. EHF group (day 10 vs. 14, *p* < 0.05) (subset analysis). Mean enteral intake significantly increased by day 14 for the IPF group (*p* < 0.05), reflecting group divergence as achieved feeding volumes increased. Conclusions: Results suggest shorter time to full enteral feeding and higher feeding volume achieved by study end in preterm infants receiving intact protein preterm formula versus extensively hydrolyzed formula.

## 1. Introduction

Meeting nutritional needs in the first weeks of life for preterm infants is a major clinical challenge within the neonatal intensive care unit (NICU) due to feeding intolerance [1]. Despite the World Health Organization (WHO) recommending mother’s own milk as the source of enteral nutrition for low-birth-weight infants and donor milk when mother’s own milk is unavailable, at point-of-care, the timing and practice of introduction to feedings varies widely [2]. Access to mother’s own or donor human milk likely accounts for these differences in practice [3]. In addition, the European Society of Pediatric Gastroenterology, Hepatology, and Nutrition (ESPGHAN) [4] and the American Academy of Pediatrics (AAP) [5] provide only general guidance on the composition of preterm infant formulas.

Over the past two decades, interest in using hydrolyzed protein formulas increased [6,7,8]. Recently, a national survey in France reported that 91% (158/174) of units routinely used hydrolyzed cow milk protein formulas (most commonly due to the absence of human milk availability) [9]. However, rigorous systematic reviews investigating the advantage of hydrolyzed protein formulas over intact protein formulas reached the conclusion that existing data do not support the use of protein hydrolysate [6,10,11]. Expanded guidance regarding recommended macro-nutrient composition of preterm formulas when required, especially use of hydrolyzed versus intact cow milk proteins, would further inform NICU practices.

Measures of gastrointestinal tolerance and daily weight gain guide feeding advancement decisions, in turn affecting the duration of parenteral nutrition and need for vascular catheters, both risk factors for nosocomial sepsis and other catheter-related complications, all of which can increase the potential of prolonged hospital stay [12,13,14,15,16]. Smooth, and hopefully rapid, advancement in feeding volumes is a short-term nutritional management goal for preterm infants that motivates strategies to achieve higher feeding volumes in order to discontinue intravenous fluids. Increased availability of clinical data for short-term feeding and gastrointestinal tolerance outcomes in preterm infants receiving intact or hydrolyzed protein preterm infant formulas, especially during the first few weeks of life when achievement of full feeding is often accomplished, could help establish improved guidelines and NICU practice.

In the current study, days to first achieving full enteral feedings (defined as a daily intake of ≥140 mL/kg/day) was the primary outcome variable compared in preterm infants receiving one of two isocaloric, marketed cow milk-based study formulas (Mead Johnson Nutrition, Evansville, Indiana) over the first 14 days of feeding: intact protein preterm infant formula (Enfamil^®^ Preterm) or extensively hydrolyzed protein infant formula (Pregestimil^®^). Secondary objectives included evaluation of measures of gastrointestinal tolerance.

## 2. Methods

### 2.1. Study Design and Participants

In this pilot, double-blinded, randomized, controlled, prospective clinical trial [17], eligible infants were enrolled between February 2014 and February 2016 in the NICU of University of Bari “Aldo Moro”, Bari, Italy. Participants were followed through hospital discharge. The research protocol and informed consent forms observing the Declaration of Helsinki (including October 1996 amendment) were approved by the “Azienda Ospedaliero-Universitaria Consorziale Policlinico” Independent Ethics Committee, Bari, Italy. The study complied with good clinical practices. Parents or legally authorized representatives provided written informed consent prior to enrolment.

Eligible infants satisfied the following criteria: 28–33 completed weeks gestational age (GA); singleton or twin birth; birth weight of ≥700 to 1750 g and appropriate for GA (AGA, defined as birth weight between and inclusive of the 10th and 90th percentiles on the Italian neonatal study growth chart [18]; enteral intake <30 mL/kg/day or never received enteral feedings. Newborns were enrolled by or before the first 24 h of enteral feeding. Exclusion criteria included the following: (1) Apgar score <4 at 5 min; (2) history of underlying metabolic or chronic disease, congenital malformation, or any other condition likely to interfere with ability to ingest food, normal growth and development, or participant evaluation; (3) major surgery requiring general anesthesia prior to or on day of randomization (patent ductus arteriosus (PDA) ligation allowed); (4) unstable blood pressure (<5 mg/kg/min dopamine allowed); (5) ventilator-dependent or requiring >40% fractional inspired supplemental oxygen (FiO2) on day of randomization (nasal cannula and/or nasal continuous positive airway pressure (CPAP) and/or oxygen hood allowed); (6) diagnosed Grade III or IV intraventricular hemorrhage (IVH) prior to or on day of randomization.

### 2.2. Randomization and Study Group Allocation

A computer-generated, randomization schedule stratified by gender and birth weight (700–1250 g and 1251–1750 g) was created by the study sponsor and provided in opaque, sealed, consecutively numbered envelopes. Following the physician’s order for first enteral feeding, one of two study formulas was randomly assigned by opening the next sequential envelope from the appropriate set at the study site: intact protein preterm infant formula (IPF) or extensively hydrolyzed infant formula (EHF) (Table 1). Twins were randomized following the same criteria used for singleton infants. Both study formulas were in opaque containers with a paper label detailing the following: brief description of product (infant formula powder); amount (g) per container; a statement of “for investigational use only, not for sale”; sponsor name and address; instructions for preparation and use; and storage requirements.

True masking of the formulas was a challenge because the study formulas smell different and, after mixing, are visually distinct. To ensure personnel responsible for monitoring the study were blinded to study product identification, a third-party individual (a head nurse not directly involved in patient care) received the sealed envelope containing the product code information, confidentially prepared and poured the product into the appropriate feeding container labeled with the product code, participant’s randomization number and initials, and date and time feeding prepared, and placed it in the appropriate NICU refrigerator until ready for use. To maintain the double-blind design, nurses involved in patient care and mothers both used opaque containers.

All mothers were encouraged to provide their own milk; study formula was supplemented as needed per randomization over the first 14 feeding days. Participant masking could be broken by study sponsor personnel in the event of a medical emergency. In this study, it was not necessary to break the study code prematurely.

### 2.3. Feeding Protocol

Parenteral nutrition (PN) with a minimum 2 g/kg/day of protein was initiated on NICU admission. The composition of PN through the study period is provided in Table A1. The protein content of PN was increased to supply a maximum of 4 g/kg/day through the study period. Trophic feeds (10–15 mL/kg/day) started within 24 h of life. Human milk feeding was encouraged; assigned study formulas were used when mother’s own milk was not available. At the attending physician’s discretion, nutritional feeds started at 15–20 mL/kg/day and were increased by 15–20 mL/kg/day, dependent upon enteral tolerance. All participants were initially fed continuously by nasogastric tube using an infusion pump. Infants could receive intermittent bottle feeding according to their ability to suck effectively. If the gastric residual was ≥50% of the previous feeding (or previous 3 h of feeding for infants on a continuous infusion), then feedings were held for 1 h and the baby was reevaluated. If the residual persisted, results of a complete blood count, C-reactive protein, and abdominal radiograph were evaluated. If normal, then feedings were continued at the same rate for 2–3 days before resuming feeding advancement. The PN was reduced as enteral nutrition was increased and discontinued when achieved enteral feeding volumes reached 140 mL/kg/day.

### 2.4. Study Outcomes

Following parental informed consent, eligible infants underwent physical examination, and birth characteristics (sex, GA, anthropometrics, mode of delivery, birth type) were collected. Feeding modalities and enteral intakes were recorded daily. Feeding tolerance measures (gastric residuals, number of stools/day, incidence of abdominal distention (defined as an increase in abdominal girth >1 cm since previous nursing assessment), regurgitation/emesis of milk (>1 mL), feedings withheld ≥4 h at a time due to intolerance, and bloody stools), and indicators of respiratory status (incidence of apnea and/or bradycardia, use of supplemental oxygen/CPAP/nasal cannula, mechanical ventilation) were collected through the study period. For each participant, daily residual volumes (expressed as a percentage of that day’s daily intake) were averaged over the study period and included only days when gastric residuals were measured and feedings were provided enterally. Accepted criteria used for feeding tolerance in the current study included gastric residual volumes <50% of the food amount given in the previous meal (or during the 3 h before if fed continuously) and absence of vomiting and abdominal distension [19]. Body weight was recorded daily. Length and head circumference were recorded once a week. Growth rates were calculated over the study period by fitting a linear regression model to each participant’s data. Adverse events (AEs) and global morbidity including incidence of early and late onset sepsis [20], necrotizing enterocolitis (NEC); using modified Bell’s staging criteria ≥2) [21], bronchopulmonary dysplasia (BDP) [22], retinopathy of prematurity (ROP) [23], extrauterine growth retardation (EUGR), and intraventricular hemorrhage (IVH) [24] were collected throughout the study. Growth that fell below the 10th percentile in comparison to a healthy, reference fetus of the same gestational age was considered EUGR at day 14 [25]. Postmenstrual age at discharge and duration of hospitalization were calculated for all participants.

### 2.5. Statistical Analysis

The feeding advancement data referenced most often (published in 2002) evaluated preterm infants receiving a hydrolyzed whey:casein (60:40) blend (*n* = 46) vs. intact protein formula (*n* = 41), which may also influence clinical outcomes [7]. To provide more up-to-date evidence regarding the potential benefits of routine use of an extremely hydrolyzed, hypoallergenic casein-based protein in a preterm NICU population through hospital discharge, new preliminary data were needed. Consequently, a pilot sample size was chosen with the expectation that 30 participants per group would complete the study, representative of infants in a standard NICU environment and a method to quickly estimate the sample size needed for a larger growth study. The day of first enteral feeding was considered study day 1. Achievement of full enteral feedings using time-to-event Kaplan–Meier estimates was compared between groups by log rank test. Daily enteral intake (mL; mean ± SD) was calculated for each participant (last study day was excluded as it could be a partial day with intake amounts recorded for <24-h period); group comparisons by study day were performed by Student *t*-test and over the study period by analysis of variance (ANOVA). Weight growth rates were analyzed by analysis of covariance with weight on study day 1 as covariate. Length and head circumference growth rates, achieved weight, length, and head circumference at study day 14, and achieved weight at hospital discharge were analyzed by ANOVA. Participant characteristics at enrolment were compared by Student *t*-test (gestational age and anthropometrics) or chi-square (gender, birth type, caesarean section, and Apgar score). Study discontinuation, serious AEs, sepsis (early and late onset), ROP, and EUGR were analyzed by chi-square test. NEC, BPD, and IVH were analyzed by Fisher’s exact test. Tolerance outcomes (percentage of days with presence of symptoms) were compared by median test. Postmenstrual age at discharge was analyzed by Student *t*-test. Duration of hospitalization was analyzed by Kaplan–Meier curves and log rank test. Populations for evaluation established per protocol included the following (1) primary: participants who met study entrance criteria and completed study feeding, and (2) subset analysis: those participants who completed study feeding and received ≥75% enteral intake from study formula (average over total study days; mL/kg/day). All observations of participants who did not reach full enteral feeding by day 14 or prior to experiencing four consecutive days with no enteral intake were treated as censored observations on the study day prior to this four-day period. All tests were two-sided and conducted at α = 0.05. All analyses were conducted using IBM^®^ SPSS^®^ Statistics 23.

## 3. Results

### 3.1. Participants

**Figure 1:**
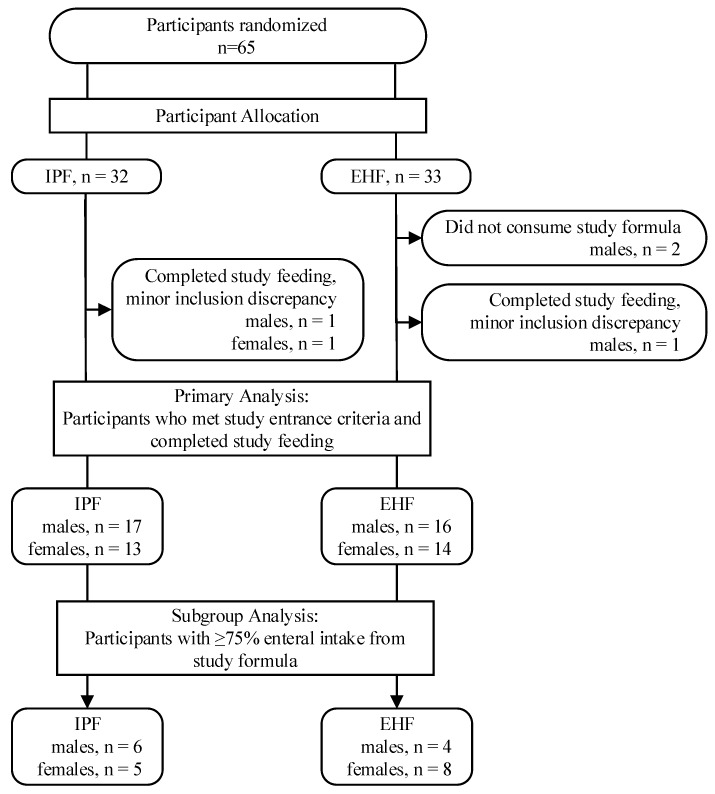
Study allocation.

Written informed consent was obtained from parents for all 65 enrolled participants (IPF: *n* = 32; EHF: *n* = 33). Two participants were randomized to the EHF group but consumed no study formula; three completed the study (and assigned study feeding) but were determined to have inclusion criteria discrepancies (IPF: *n* = 1, EHF: *n* = 2) (Figure 1) [26]. These five participants were not included in subsequent statistical analyses. A total of 60 participants completed the study (IPF: 30; EHF: 30) and were included in the population for primary analysis; of these participants, 23 received ≥75% enteral intake from study formula (IPF: *n* = 11; EHF: *n* = 12; *p* = 0.79) and comprised the population for subset analysis. Birth anthropometric measures, as well as gender, birth type, Apgar score, and gestational age (at birth), were similar between groups (Table 2) [26].

### 3.2. Feeding Advancement

**Figure 2:**
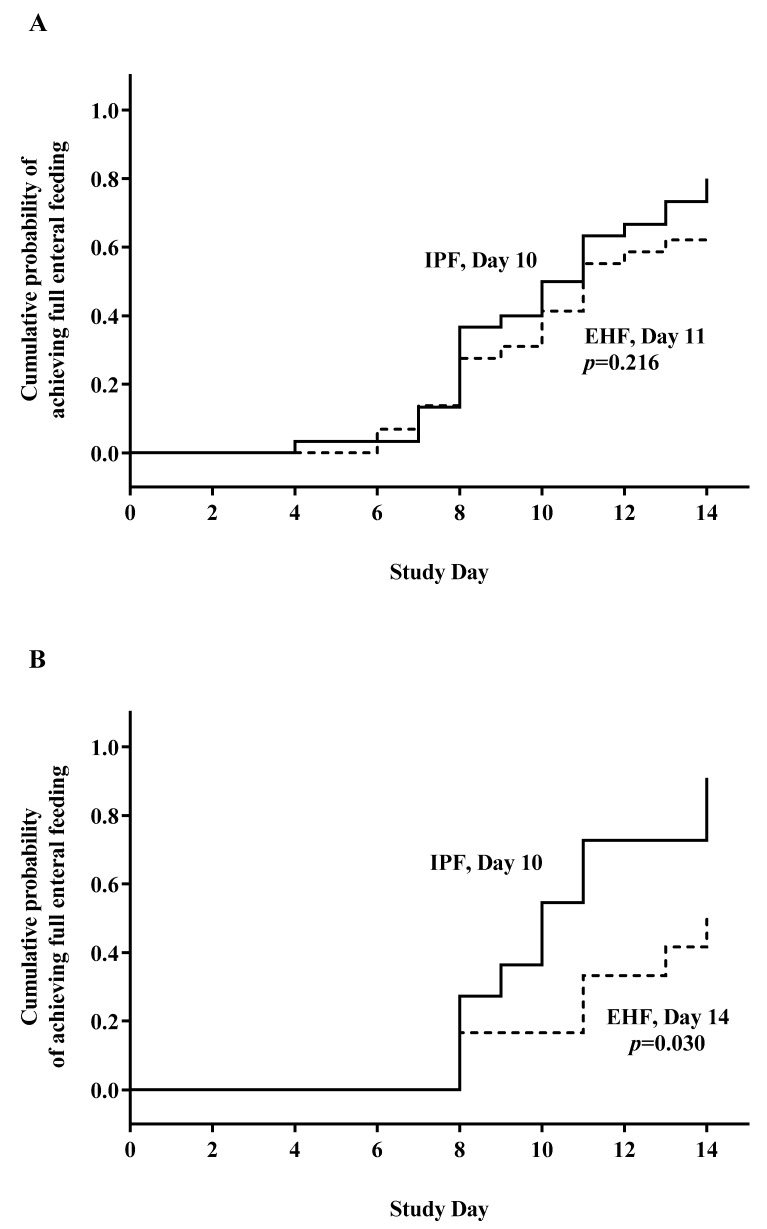
Days to full enteral feeding (≥140 mL/kg/day): (**A**) No significant difference for the intact protein preterm formula (IPF) vs. extensively hydrolyzed formula (EHF) group for all who met study entrance criteria and completed study feeding (day 10 vs. 11; *p* = 0.216). (**B**) Subset analysis: significantly shorter for the IPF vs. EHF group for participants receiving ≥75% enteral intake from study formula intake (day 10 vs. 14; *p* = 0.030).

**Figure 3:**
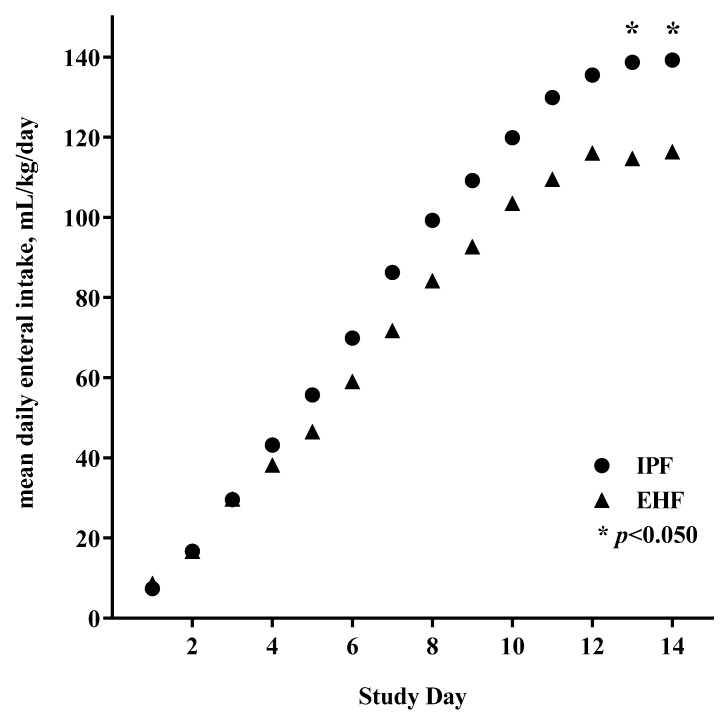
Mean daily enteral intake (mL/kg) began to diverge by study day 4; * significantly greater for the IPF (●) vs. EHF (▲) group by day 13 (*p* = 0.042) and continued at day 14 (*p* = 0.047).

Full enteral feeding was achieved in the first 14 days by 43 participants (72%). Median time to achievement of full enteral feeding was similar between groups overall (IPF: day 10; EHF: day 11; *p* = 0.216) (Figure 2A) but significantly shorter for the IPF vs. EHF group (day 10 vs. 14; *p* = 0.030) for participants receiving ≥75% study formula intake (Figure 2B). Achievement of full enteral feeding by day 14 was similar between groups overall (IPF: 24/30, 80%; EHF: 19/30, 63%; *p* = 0.152), but higher in the IPF group for participants receiving ≥75% study formula intake (IPF: 10/11, 91%; EHF: 6/12, 50%; *p* = 0.045). No gender or group differences were detected in mean enteral intake (mL/kg/day) over the study period (primary analysis). By study day, significantly higher mean enteral intake emerged by study end for the IPF vs. EHF group (day 13, *p* = 0.042; day 14, *p* = 0.047), reflecting greater divergence between study groups as achieved feeding volumes increased (Figure 3).

### 3.3. Growth

No significant group differences in weight, length, or head circumference growth rates were detected (primary or subset analysis, data not shown). No significant group differences in achieved weight, length and head circumference at study day 14 or at hospital discharge were detected (primary or subset analysis, data not shown).

### 3.4. Tolerance Measures, Indicators of Respiratory Status, and Global Morbidity

No group differences in tolerance measures, indicators of respiratory status, and global morbidities (incidence of EOS, LOS, BPD, IVH, ROP, or EUGR) were detected (primary or subset analysis) (Table 3). No episodes of NEC or other serious AEs were reported. No group differences in postmenstrual age at discharge and length of hospitalization were detected (primary or subset analysis).

## 4. Discussion

In the current pilot study, shorter time to full enteral feeding and higher achieved feeding volume by study end were demonstrated in preterm infants receiving an intact protein preterm formula compared to an extensively hydrolyzed infant formula during the first 14 days of feeding. Mean daily enteral intake (mL/kg) appeared to diverge by study day 4 for the intact protein formula group with a significant increase emerging by study end as feeding volumes increased. No episodes of NEC were reported. No group differences in tolerance measures, incidence of AEs, or positive cultures for sepsis were detected, confirming that both intact protein preterm formula and extensively hydrolyzed formula are safe and well tolerated in preterm infants during a NICU stay.

As noted, little published evidence to date supports better tolerance associated with protein hydrolysate formulas vs. intact protein preterm formulas. In the often-cited Mihatsch et al. (2002) study (*n* = 87), preterm infants randomized to receive a hydrolyzed formula vs. a standard preterm formula achieved full enteral feeding earlier (10 vs. 12 days), but no group differences were reported in any feeding intolerance measurements (gastric residuals, mean gastric residual volume, or proportion of gastric residuals >5 mL/kg body weight [BW]) [7].

Only a retrospective analysis in a Chinese population (*n* = 692) reported significantly lower incidence of feeding intolerance and faster achieved complete intestinal feeding time (12 vs. 14 days) for preterm infants receiving extensively hydrolyzed whey formula designed for preterm infant vs. standard preterm formula [27].

In accordance with our results, a 2009 study (*n* = 80) demonstrated no improvement in tolerance or enteral intake for preterm infants receiving partially hydrolyzed whey vs. intact whey/casein preterm infant formula [28]. Furthermore, a large randomized trial in a Chinese infant population (*n* = 328) showed that preterm infants (<32 weeks gestation) fed a standard preterm formula vs. an extensively hydrolyzed formula achieved 50 kcal/kg significantly faster (12 vs. 16 days), weaned from PN significantly sooner (18 vs. 24 days), and were discharged from the hospital significantly sooner (29 vs. 36 days) [29].

It is important to note that lower protein and calcium bioavailability, poorer growth, and lower anabolic biomarkers were demonstrated in hydrolyzed formulas compared to isonitrogenous intact protein formulas [30]. Consequently, demonstration of comparable growth requires increased hydrolyzed protein concentrations (~10%) and the provision of ~25% more calcium [31,32,33,34]. Higher amounts of protein and calcium added to compensate for lower bioavailability include higher formula osmolality, which—when excessive—may increase risk of NEC or direct mucosal injury based on a series of older case reports [35].

Finally, we are aware that one of the limitations of this pilot study is that it included a large percentage of infants primarily receiving human milk. However, the active practice of breastfeeding implementation at the study site provided an opportunity to assess study outcomes within the context of real-world NICU practices and the use of mixed feeding.

## 5. Conclusions

In this pilot study, a statistically significant shorter time to full enteral feeding and higher achieved feeding volume by study end were demonstrated in preterm infants predominantly fed an intact protein preterm formula compared to an extensively hydrolyzed infant formula during the first 14 days of feeding.

Current data, which suggest the continued use of intact protein preterm infant formulas designed to meet the specific macro- and micro-nutrient needs of preterm infants when human milk is unavailable, also emphasize the need for a larger, well-designed, prospective, masked RCTs to guide expert recommendations and neonatal unit best practices for use of formulas with hydrolyzed versus intact cow milk proteins.

## Figures and Tables

**Table 1:** Study formulas.

Characteristics	Study Formula *	
	IPF	EHF
Nutrient density (cal/fl oz)	24	24
Nutrient composition per 100 kcal		
Total Protein (g, % calories) ^†^	3.0, 12%	2.8, 11%
Total Carbohydrate (g, % calories) ^‡^	11, 44%	10.2, 41%
Total Fat (g, % calories) ^§^	5.1, 44%	5.6, 48%
Arachidonic acid (ARA), mg	34	34
Docosahexaenoic acid (DHA), mg	17	17
Potential Renal Solute Load (mOsm/100 Cal)	27	25
Potential Renal Solute Load (mOsm/100 mL)	21	21
*Osmolality* (*mOsm/kg H_2_O*)	310	340
*Osmolarity* (*mOsm/L*)	270	300

* Marketed study formulas (Mead Johnson Nutrition, Evansville, IN, USA) included intact cow milk protein preterm formula (IPF: Enfamil^®^ Preterm) and extensively hydrolyzed cow milk protein formula (EHF: Pregestimil^®^). ^†^ Sources of protein, IPF: skim milk and whey protein concentrate; EHF: casein hydrolysate, l-cystine, l-tyrosine, l-tryptophan. ^‡^ Sources of carbohydrate, IPF: glucose polymers and lactose; EHF: corn syrup solids and modified corn starch. ^§^ Sources of fat: medium-chain triglyceride (MCT) oil (38%, IPF; 53%, EHF), soybean oil, high oleic sunflower oil, coconut oil (IPF only), lecithin, and ascorbyl palmitate; ARA and DHA from single-cell oils.

**Table 2:** Infant characteristics at study entry.

Infant Characteristics	Study Group		*p*
	IPF	EHF	
Gender, *n* (%)			
Male	17 (56.7)	16 (53.3)	0.795
Female	13 (43.3)	14 (46.7)	
Birth type, *n* (%)			
Singleton	15 (50)	17 (56.7)	0.605
Twin	15 (50)	13 (43.3)	
Caesarean Section, *n* (%)	26 (86.7)	26 (86.7)	1.000
Apgar score, *n* (%)			
6–7	3 (10.0)	6 (20)	0.760
8	6 (20.0)	6 (20)	
9	16 (53.3)	12 (40)	
10	5 (16.7)	6 (20)	
Gestational age (days) *	30.1 (1.6)	30.9 (1.9)	0.803
Birth anthropometrics *			
Weight (g)	1278.7 (259.7)	1301 (293.2)	0.756
Length (cm)	38.1 (2.8)	38.1 (3.3)	0.973
Head circumference (cm)	27.3 (1.7)	27.7 (2.4)	0.481

* Mean ± standard deviation (SD).

**Table 3:** Tolerance measures, respiratory status, and global morbidity during the study period and demographics at hospital discharge by primary (all participants who completed the study per protocol) and subset analysis (participants who received ≥75% enteral intake from study formula).

Tolerance Outcomes	Primary Analysis		*p*	Subset Analysis		*p*
	IPF, *n* = 30	EHF, *n* = 30		IPF, *n* = 11	EHF, *n* = 12	
Number of stools/day *	2 (0.8)	2 (0.9)	0.525	1 (0.5)	2 (0.9)	0.160
Abdominal distention **	0	0	1	0	0	1
Regurgitation/emesis **	21	18	1	29	18	0.684
Feedings withheld ≥4 h **	14	25	0.060	14	25	0.400
Bloody stools **	0	0	0.472	0	0	1
Parenteral nutrition, days *	11 (7.1)	15 (12.3)	0.181	10 (6.5)	15 (12.6)	0.315
Global morbidities, *n* (%)						
Early-onset sepsis	2 (6.7)	3 (10)	0.640	1 (9.1)	1(8.3)	0.739
Late-onset sepsis	6 (20)	6 (20)	1.000	2 (18.2)	3 (25)	0.545
BDP	0 (0)	3 (10)	0.119	0 (0)	1 (8.3)	0.522
IVH	1(3.3)	4 (13.3)	0.177	0 (0)	0 (0)	1.000
ROP	5 (16.7)	7 (23.3)	0.519	1 (9.1)	4 (33.3)	0.185
EUGR	18 (60)	16 (53.3)	0.602	8 (72.7)	9 (75)	0.635
Hospitalization, days ^†^	44 (38–50)	49 (34–64)	0.236	49 (36–62)	44 (28–60)	0.871
Postmenstrual age at discharge, weeks *	37 (2.1)	38 (2.8)	0.229	37 (2.1)	38 (1.9)	0.740
Weight at discharge, g *	2141 (391)	2140 (283)	0.996	2176 (293)	2128 (204)	0.704

* Mean (SD). ** Median percentage of days with presence of symptoms. ^†^ Median (95% confidence interval (CI)). BDP—bronchopulmonary dysplasia; IVH—intraventricular hemorrhage; ROP—retinopathy of prematurity; EUGR—extrauterine growth retardation (EUGR).

## Appendix A

**Table A1:** The composition of PN through the study period.

Day of Life	1	2	3	4	5	6	7	Max.
Calories (k/kg/day)	40	50	65	75	85	90	100	110
Glucose (g/kg/day)	6	7	8	9	10	11	12	14
Lipids (g/kg/day)	0.5	1.0	1.5	2.0	2.5	3.0	3.0	3.5
Proteins (g/kg/day)	2.0	2.5	3.0	3.5	3.5	3.5	3.5	4.0
Calcium (mg/kg/day)	40	50	60	65	65	65	65	70
Phosphorus (mg/kg/day)		20	30	40	45	45	45	55
Magnesium(mg/kg/day)			5	5	5	5	5	10
